# Transnational Issues in Quarantine

**DOI:** 10.3201/eid1009.040281

**Published:** 2004-09

**Authors:** Cleto DiGiovanni, Jerome Conley, David Hamon, Meade Pimsler

**Affiliations:** * Defense Threat Reduction Agency, Fort Belvoir, Virginia, USA;; † The Institute for Crisis, Disaster, and Risk Management of The George Washington University, Washington, DC, USA;; ‡ Northrop Grumman Information Technology, Alexandria, Virginia, USA

**Keywords:** conference summary, biological terrorism, infection control, public health, quarantine, risk communication

How to promote transnational collaboration in implementing quarantine was the topic of a January 19–23, 2004, conference sponsored by the Defense Threat Reduction Agency. Fifty invited participants discussed the status of quarantine planning in 13 countries (the Americas, Israel, and several members of the European Union [EU] nations). Held in the Wilton Park Conference Centre, Sussex, United Kingdom, the conference, "Quarantine following an International Biological Weapons Attack: Building Cooperation, Achieving Consistency," also addressed quarantine in response to emerging infectious diseases.

Participants first examined the legal foundation for quarantine in their countries. Federal Canadian quarantine law applies only to national ports of entry or exit; provincial laws govern quarantine in the provinces. The U.S. Centers for Disease Control and Prevention has quarantine responsibilities at national ports of entry or departure; this agency may also become involved when a disease is spreading across state borders or even within a state (when invited by the governor of the state or ordered by the U.S. Secretary of Health and Human Services or the U.S. President). In general, however, quarantine in the United States is a local or state government issue. Quarantine laws in these jurisdictions vary, and some public health authorities expressed reluctance to address their shortcomings through legislation for fear that skeptics of quarantine would further weaken the laws. Other nations would turn to the World Health Organization and its International Health Regulations of 1969 (IHR) for guidance. A revised IHR should be available by 2005, but currently it lists only three diseases—plague, cholera, and yellow fever—as subject to quarantine and offers scant help in planning quarantine. Thus, the legal framework for quarantine varies and contributes little to the construction of a consistent approach to quarantine among nations.

European public health officials have forged some bilateral cooperative agreements and are discussing establishing a regional disease control center for EU nations. They are not, however, developing and testing national or transnational plans for possible large-scale quarantine. Some participants thought that consistency in developing and implementing quarantine measures was not necessarily desirable, given that each nation must deal with threats in accordance with its own culture, laws, and traditions. Others thought that inconsistencies in response to the same disease threat might encourage persons to question the need for quarantine measures and choose not to comply. The United States also has not developed comprehensive quarantine plans, trained staff, or conducted quarantine exercises in local communities, despite recently issued federal quarantine guidelines. Especially lacking are processes and procedures to clarify decision-making and coordination in communities with multiple jurisdictions.

The heightened concern of the United States about bioterrorism was not shared by others at the conference, although all agreed that persons would likely demand a federal response to a health crisis caused by terrorists, including any required quarantine. Other issues discussed included assurances of compensation for income lost while in quarantine (strongly recommended as a component of any quarantine plan) and psychosocial support to reduce the sense of isolation experienced by many persons while in quarantine. Officials with information management experience during health- and nonhealth-related crises commented on the need for caution in making public statements when faced with a new and evolving threat.

The conference permitted participants to establish working relationships with one another, but it also highlighted gaps in comprehensive transnational quarantine planning. The complete conference report is available at http://www.dtra.mil/about/ASCO/wpc/wpc.cfm

